# OSTEOSARCOPENIA: A GEROSCIENCE APPROACH

**DOI:** 10.1093/geroni/igae098.0322

**Published:** 2024-12-31

**Authors:** Gustavo Duque

**Affiliations:** Bone, Muscle & Geroscience Group, Research Institute of the McGill University Health Centre, Montreal, Quebec, Canada

## Abstract

In older persons, the combination of osteopenia/osteoporosis and sarcopenia - known as osteosarcopenia - has been proposed as a subset of individuals at higher risk of adverse outcomes. The pathophysiology of osteosarcopenia results from a complex set of interactions between bone, muscle, and fat influenced by genetic, mechanical, and biochemical factors. There is a complex crosstalk system between muscle and bone, in which fat has become a new player. This communication system comprises osteokines, myokines, adipokines and other secreted factors (i.e., extracellular vesicles, exosomes and microRNAs), some of which have positive or negative effects on muscle and/or bone metabolism and function. These effects include alterations in most of the hallmarks of aging, triggered by conditions such as sedentarism, obesity, malnutrition, inflammation, and menopause, which induce tissue loss and dysfunction. In contrast, anabolic factors could be stimulated to prevent muscle and bone loss of mass and function. Therefore, osteosarcopenia is an optimal condition in which geroscience-based approaches could target several hallmarks of aging to have a beneficial dual effect on bone and muscle. This session will discuss the biological characteristics of osteosarcopenia shared by muscle and bone, their relevance in the pathogenesis of the condition, and their potential use as therapeutic targets in osteosarcopenia.

